# Evaluation of Oral Health Status and Oral Care of Patients with Rheumatoid Arthritis

**DOI:** 10.1155/2020/8896766

**Published:** 2020-10-30

**Authors:** Małgorzata Radwan-Oczko, Irena Duś-Ilnicka, Pamela Richards, Anna Marie Thomsen, Claus Rasmussen

**Affiliations:** ^1^Department of Oral Pathology, Wrocław Medical University, ul. Krakowska 26, 52-425 Wrocław, Poland; ^2^Patient Research Partner, University of the West of England, Bristol, UK; ^3^Rheumatology Department, Clinic of Medicine, North Denmark Regional Hospital, Hjoerring, Denmark

## Abstract

**Objective:**

Rheumatoid arthritis (RA) and periodontal disease (PD) are common chronic, immunoinflammatory, destructive, and progressive diseases; however, the correlations between those two are not yet widely discussed. The purpose of this study was to evaluate the relationship between the selected demographic and clinical parameters of RA patients and oral health status parameters, on the basis of self-assessment.

**Materials and Methods:**

Three hundred patients under treatment were included in the study. Questionnaires were completed by 164 out of 300 patients.

**Results:**

A total of 100 females and 64 males took part in the study, with a mean age of 65 ± 11.1 years. In younger patients, the disease activity score (DAS28) was higher, and it was associated with pain or discomfort in the oral cavity and with difficulties in toothbrushing. Discomfort or pain in the oral cavity was to a significant extent associated with the poor gingival state, gingival bleeding, and difficulties in biting or chewing.

**Conclusions:**

In RA patients, difficulties in biting or chewing, discomfort or pain in oral cavity, feeling of the presence of movable teeth, and gingival bleeding are indications of periodontal infection. Maintaining awareness of oral health and RA is a key issue in the simultaneous management of proper oral care and RA due to the mutual influence of those two factors.

## 1. Introduction

The relation between oral health and general health is currently widely described and documented [1, 2]. In several studies, oral infection with periodontal disease (PD) has been associated with systemic conditions, such as diabetes, cardiovascular disease, pulmonary infection, liver diseases, strokes, ankylosing spondylitis, and rheumatoid arthritis (RA) [3, 4]. That connection was demonstrated in epidemiological studies and systemic reviews. Patients suffering from the aforementioned general diseases presented poor oral hygiene and/or poor dental and periodontal condition, with parameters such as bleeding, gingival inflammation, and depth of periodontal pockets more often statistically significant than in healthy control groups. Furthermore, clinical studies have revealed that improvements in oral hygiene and initial nonsurgical periodontal therapy can result in the reduction of severity of systemic conditions [1–7].

Both PD and RA are common chronic, inflammatory, destructive diseases. PD, as a bacterial infection-related disease, is characterized by gingival inflammation, periodontal pocket formation, attachment loss, alveolar bone loss, and finally, tooth loss. Recently, it has been found that the gingival bacteria, *Aggregatibacter actinomycetemcomitans*, may cause citrullination and, therefore, possibly trigger RA [8]. In RA, autoimmune pathogenesis is indicated; however, the exact cause is still not known. The disease affects the peripheral joints with characteristic symptoms of chronic pain and swelling, destruction of the cartilage and bone, and finally, functional disability. High prevalence of periodontitis in RA patients, from 51% to even 94%, at different levels of severity, was described in many epidemiological studies [9, 10]. Hence, the new approach to the subject of relation between those diseases is valid. Disease activity score (DAS) used in this study was first developed in 1990s to evaluate patients in remission and those with all stages of the disease activity: from low, moderate, to severe disease activity. The index serves to quantify disease activity in RA in daily clinical practice. Since that tool is widely accepted for the evaluation of patients' condition, it is also used in clinical trials and in the long-term observational studies. DAS index provides information about the numbers of swollen and tender joints, acute phase response, and patient self-report referring to general health status by the time of evaluation [11]. The aim of the study was to evaluate the relation of lifestyle factors to clinical and oral health parameters on the basis of RA patients' self-reported assessment and their recorded DAS28.

## 2. Materials and Methods

The outpatient clinic where the present study was carried out cares for 1,200 patients with RA. The patients are examined twice a year, and the disease activity score based on DAS28 is calculated and recorded. A DAS28 score of less than 2.6 indicates remission in RA patients. All the patients are registered in the clinical database. The patients are treated according to EULAR guidelines [12], and both the expensive medicines and the consultations are paid by the public hospital. Questionnaires were emailed to a random sample of 300 patients, and 164 (55%) completed answers have been received. The questionnaire is accessible as an electronic supplement. Since 1950s, public dental care in Denmark has been free for children up to 18 years of age. For adults, there is a differentiated level of reimbursement of dental care costs. Additionally, the authors state that since the Danish Ethic Committee do not accept applications for the questionnaire studies, the study was approved by the Danish Data Protection Agency (ID 2017-47). For the purpose of this study, all protection and anonymization processes of the fragile data were provided for the questioned patients.

## 3. Results

### 3.1. Statistical Methods

Depending on the distribution of variables, the continuous parameters were expressed as the number of cases (*N*), mean, and standard deviation (SD) or as the number of cases (*N*), median (*M*), range (min-max), and lower and upper quartiles (25 Q–75 Q). Statistical significance between means for different groups was calculated using one-way analysis of variance (ANOVA) or alternatively with a nonparametric test (the Mann–Whitney *U* test for two groups and the Kruskal–Wallis rank sum test for more groups) when the variances in groups were not homogeneous. Homogeneity of variance was determined with the use of Bartlett's test. Statistical significance between frequencies was calculated using the chi-square test (*χ*_d*f*_^2^) incorporating Yate's correction with the corresponding degree of freedom (d*f*) (d*f*=(*m* − 1)*∗*(*n* − 1), where *m* is the number of rows, *n* is the number of columns).

The relation between the two parameters was assessed using correlation analysis. Pearson correlation coefficients were calculated. A *p* value of less than 0.05 was required to reject the null hypothesis. Statistical analysis was performed using EPIINFO Ver. 3.4.3 (08-11-2007) software package.

### 3.2. Patients Data Analysis

A total of 100 females and 64 males took part in the study, with a mean age of 65 ± 11.1 years. The frequency of investigated parameters including lifestyle factors is presented in Table 1.

**Figure 1:**
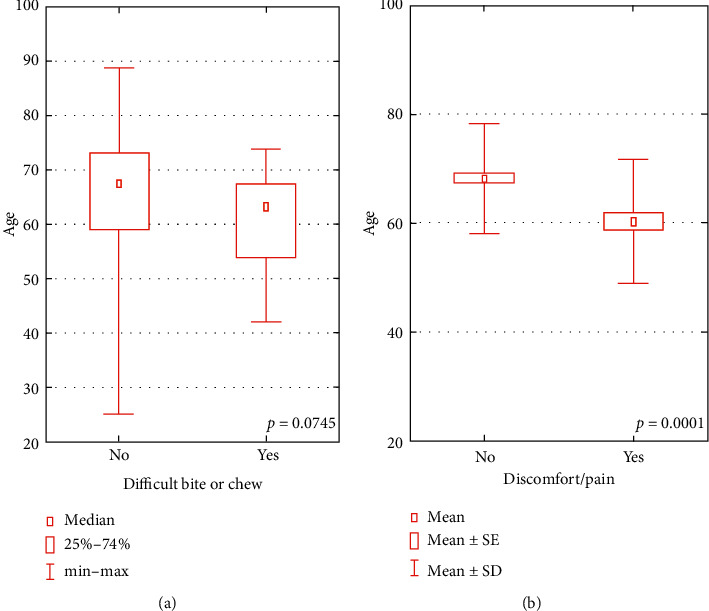
Relationship between the age and difficulties in biting or chewing and between the age and present discomfort or pain.

A dependence tendency was observed between the age and declared difficulties in biting or chewing, and a significant relationship was found between the age and declared discomfort or pain in the oral cavity within the last 6 months before the investigation (Figure 1). Presented difficulties were more often reported by younger subjects.

**Figure 2:**
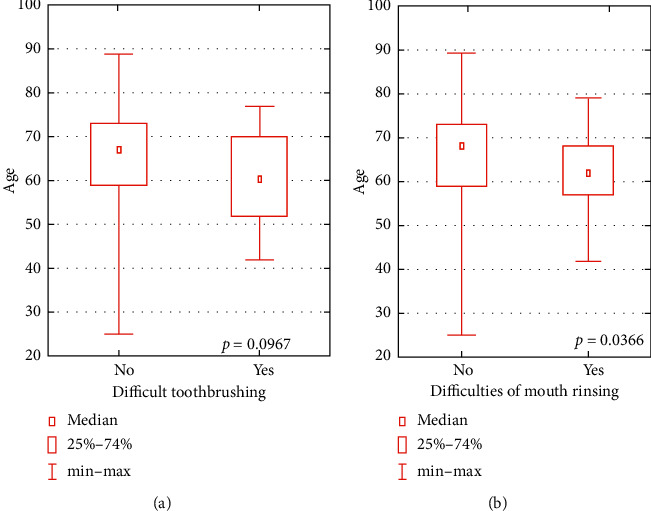
Relationship between the age and toothbrushing difficulties and between the age and difficulties in mouth rinsing.

Similarly, dependency was also found between the age of participants and difficulties in toothbrushing, and the significant relationship was observed between the age and difficulties in mouth rinsing. Moreover, both problems were significantly more often reported by younger RA patients (Figure 2).

The 12% of patients who declared current active smoking was significantly younger than nonsmoking patients (*p*=0.02). Active smokers significantly more often indicated difficulties in biting or chewing (Table 2).

**Figure 3:**
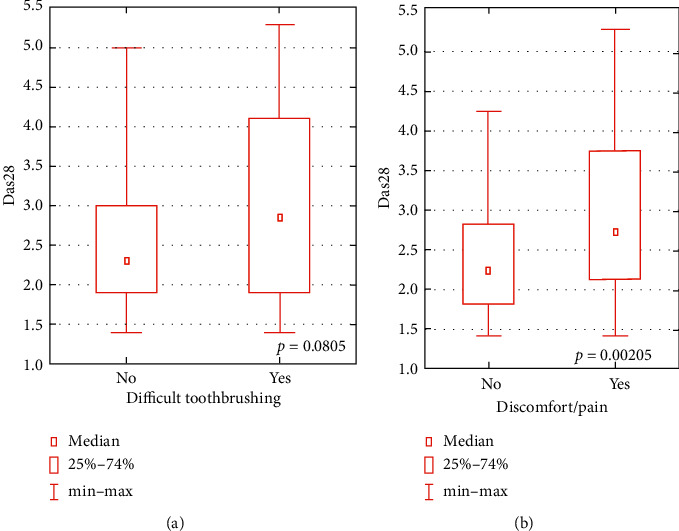
Level of DAS28 in relation to difficulty in toothbrushing and to discomfort or pain in the oral cavity.

The average value of DAS28 was 2.31 ± 0.83, and a significantly higher DAS level in linear correlation was related to the younger age of patients (*p*=0.046). The higher level of DAS28 was also significantly more often associated with the patients' discomfort or pain in the oral cavity within the last 6 months before the investigation. A pronounced but nonsignificant dependence tendency between the higher DAS28 level and difficulty in toothbrushing (Figure 3) was also observed.

The average duration of the disease from the diagnosis was 13.9 ± 11.2 years. The patients reporting difficulty in toothbrushing suffered from RA for a statistically longer time (*p*=0.01). When it comes to the status of their teeth and gingiva, it was described as good by 46% and 49% of patients and as poor by 13% and 11% of patients, respectively. 40% of patients estimated the state of their gingiva as excellent.

There were strong dependencies observed between discomfort or pain and the following parameters: worse gingival status (*p*=0.12), presence of spontaneous bleeding (*p*=0.03), presence of bleeding during or after tooth brushing (*p* ≤ 0.001), and feeling of movable teeth (*p*=0.01). This relationship between discomfort or pain and the presence of difficulties in biting or chewing was also statistically significant (Table 3). The patients who declared the presence of discomfort or pain significantly more often presented a poor gingival state, spontaneous bleeding, provoked bleeding, and difficulties in biting or chewing. However, the presence of discomfort or pain was not significantly associated with the successive factors: difficulties in toothbrushing (*p*=0.76), daily frequency of toothbrushing (*p*=0.54), and a type of toothbrush used (*p*=0.73).

In the statistical analysis, there were no dependencies found between the patients' estimated gingival status and difficulties in toothbrushing (*p*=0.63), daily frequency of toothbrushing (only one time daily or 2 and more) (*p*=0.83), and type of toothbrush used (manual used by 49% vs. electric used by 51%) (*p*=0.12). There was only a dependence between the estimated gingival status and the difficulties in mouth rinsing. Patients describing that inconvenience more often indicated a poor status of gingiva, but it was not statistically significant.

Similarly, no statistically significant dependences were found between the teeth status and the presence of toothbrushing difficulty (*p*=0.91) and rinsing mouth difficulty (*p*=0.31).

In turn, 14% of respondents reported the feeling of the presence of “movable teeth.” That symptom was significantly correlated with the oral status and was more often present in patients with the poor dental (*p* ≤ 0.001) and gingival (*p* ≤ 0.001) state.

## 4. Discussion

This cross-sectional survey describes the oral health status and oral care parameters in representative Danish patients with RA. The results showed that difficulties in biting or chewing and difficulties in toothbrushing were more commonly reported by younger patients. Moreover, difficulties in mouth rinsing and the presence of discomfort or pain in the oral cavity were associated with a younger age in a statistically significant way. It is surprising that younger patients more often reported problems concerning that daily routine activity and oral cavity care. However, on the other hand, there was an association between a higher DAS28 level and the patients' younger age. An explanation could be that younger patients in general have better dental status which requires more self-care compared to older patients who more often use prostheses, which requires less demanding self-care. In addition, a higher level of DAS28 was significantly associated with the presence of pain or discomfort in the oral cavity, and there was a dependence tendency concerning the high DAS28 level and difficulty in toothbrushing, probably because of impaired wrist and hand grip function. Equally, patients with difficulties with toothbrushing statistically longer suffered from RA.

Active smokers were statistically younger, while active smoking was significantly more often associated with difficulties in biting or chewing but not with other clinical or behavioral parameters. Both retrospective and prospective studies have demonstrated that smoking is one of the major environmental factors in RA development. Smoking has been observed to induce citrullination of peptide antigens in the lungs and has been reported to be an important factor for the development of RA in the ACPA-positive subset [13, 14]. It must be underlined that smoking is also an independent and well-established risk factor in the initiation, extent, and severity of PD. The mechanism is primarily related to downregulation of anti-inflammatory factors and upregulation of proinflammatory cytokines. A clinical symptom of inflammation—gingival bleeding—is decreased; however, deeper periodontal pockets in smokers indicate increased severity of the periodontitis [15–17]. Therefore, the lack of relationship between the smoking habit and DAS28 level or bleeding in our study might be related to the findings described in literature.

In our study, only 13% of respondents described their teeth status as poor, while 40% reported it as excellent. Similarly, gingiva status was reported as poor by 11% of respondents and as excellent by 40% of respondents. The following parameters such as poor gingival status, presence of spontaneous and provoked bleeding, presence of movable teeth feeling, and presence of difficulties in biting or chewing were significantly correlated with the reported pain or discomfort in the oral cavity. However, it was seen that these evaluated parameters were not statistically associated with the patients' problems concerning toothbrushing. Neither daily toothbrushing frequency nor the type of toothbrush influenced those clinical parameters.

The oral cavity is colonized by hundreds of different bacterial species. [18] If the growing bacterial biofilm is not removed regularly, it can be a cause of bacteria leading to an infection in other body tissues and overall health deterioration [19]. However, plaque control through exact toothbrushing and oral rinsing is sufficient for proper oral hygiene. This is even more important in RA patients treated with immunosuppressive drugs. In general, the accepted and sufficient daily routine home oral care should involve toothbrushing twice a day [20]. However, interesting findings were published showing that no significant differences between toothbrushing performed once or twice a day (every 24 or 12 hours) were observed in the gingival index level describing the gingival state and in plaque index describing the amount of dental plaque. The cited study by de Freitas et al. [21] clearly explains the lack of differences in frequency of toothbrushing and periodontal parameters. When the effectiveness of manual toothbrushes versus powered ones is considered, according to the study of systemic review of 29 short-term trials and 10 long-term trials of gingivitis scores, no statistically significant differences were found between those two types of toothbrush. In conclusion, the authors emphasize the fact that powered toothbrushes (with rotation and oscillation function) reduced plaque and gingivitis as effectively as manual toothbrushes [22]. On the other hand, it should be reminded that in case of patients suffering from RA, due to the very often present impaired hand grip function, powered toothbrushes should be recommended for home oral care. Such type of toothbrush can be more conveniently used in daily routine.

Difficulties in mouth rinsing were declared by 17% of RA patients. That symptom could be related to problems in temporomandibular joints (TMJ). However, it is only an assumption because it was not confirmed by additional examination. There are studies regarding damage of TMJ in the course of RA, but TMJ involvement in inflammatory arthritis is underestimated. Among the rheumatologists, both diagnosis of that condition and its treatment is limited. In the work of Aliko et al. [23], the most frequent symptoms of TMJ in RA were sounds and pain, whereas difficulty and limitations in mouth opening was not very often detected. In other studies, pain on mouth opening and chewing/biting are listed as typical findings [19, 24]. Therefore, respecting the frequency and severity of TMJ involvement in RA patients, this topic seems to be important in the future study.

Furthermore, only the dependence tendency between the presence of difficulties in mouth rinsing and poor gingiva rate was found. On account of the fact that those difficulties were declared by only 17% of patients and poor gingiva was reported by 11% of patients, the groups were small, and there was no possibility to prove a relationship between these parameters. The feeling of the presence of movable teeth was significantly often related to both poor dental and gingival status described by the participants. This last relationship seems to be very important, and it indicates the awareness of patients about their real oral health status associated with additional problems.

The data available from the research papers show real improvement in RA when combined periodontal and routine RA therapies are used. [25] It is worth emphasizing that RA can affect the oral health because of inability of patients to maintain proper oral hygiene. However, on the other hand, worse oral hygiene related to those difficulties results in the accumulation of bacterial plaque and development of inflammation, which may influence RA activity and treatment [26].

To the best of our knowledge, research based on RA patients' self-assessment of oral health and oral care parameters in relation to the chosen demographic and clinical factors has not yet been published. More research studies are needed to confirm our findings.

### 4.1. Conclusion and Significance of the Study

Current evidence indicates a link between oral health, general health, smoking, and development of RA. Therefore, it is of vital importance to incorporate proper oral hygiene and smoking cessation programmes and treatments in care for patients with RA.

Our study indicates that many RA patients have problems with oral hygiene and their dental self-care. In particular, younger patients reported problems with dental self-care and poor self-rated oral health. For better overall health of patients, rheumatologists can provide information about the importance of maintaining good oral health and encourage regular dental control and cessation of smoking.

## 5. Conclusion

The study among patients with rheumatoid arthritis showed that symptoms and signs indicating periodontal infection was common. Problems with toothbrushing, biting, and chewing were the most pronounced in younger patients with higher disease activity.

## Figures and Tables

**Table 1:** The frequency of the presence of investigated parameters.

Evaluated parameter	Yes (%)	No (%)
Current smoking habit	12	88
Difficulties in biting or chewing	10	90
Difficulties in toothbrushing	9	91
Difficulties in mouth rinsing	17	83
Discomfort or pain in the oral cavity	32	68
Only one toothbrushing daily	14	86
Manual vs. electric toothbrush use	49	51
Feeling of movable teeth	14	86
Spontaneous bleeding	14	86
Provoked bleeding	49	51

**Table 2:** Significant dependence between the smoking habit and difficulties in biting or chewing.

Smoking habit	No difficulties in biting or chewing	Difficulties in biting or chewing
Nonsmokers	97.3	2.7
Active smokers	84.2	15.8
Smokers in the past	83.1	16.9

*χ*_2_^2^=8.60. *p*=0.013.

**Table 3:** Dependence between the presence of discomfort or pain and presence of difficulties in biting or chewing.

Discomfort or pain	Difficulties in biting or chewing (%)	No difficulties in biting or chewing (%)
No	6.3	93.7
Yes	17.7	82.3

*χ*_2_^2^=5.05. *p*=0.024.

## Data Availability

The data used to support this study are included within this article.
